# NQO1 protects against clioquinol toxicity

**DOI:** 10.3389/fphar.2022.1000278

**Published:** 2022-10-04

**Authors:** Jamuna Chhetri, Jem Dilek, Noel Davies, Glenn Jacobson, Robert Dallmann, Nuri Gueven

**Affiliations:** ^1^ School of Pharmacy, University of Tasmania, Hobart, TAS, Australia; ^2^ Central Science Laboratory, University of Tasmania, Hobart, TAS, Australia; ^3^ Division of Biomedical Science, Warwick Medical School, University of Warwick, Coventry, United Kingdom

**Keywords:** clioquinol, oxidative stress, mitochondrial dysfunction, toxicity, neurotoxicity

## Abstract

Clioquinol (CQ) was widely used as oral antibiotic before being taken off the market in many countries in 1970, after it was linked to subacute myelo-optic neuropathy (SMON) in Japan, leading to vision loss with many patients left wheelchair-bound. The common pathology of CQ-associated SMON was reproduced in animals but none of the proposed modes of toxicity explained the restriction of CQ-induced SMON to Japan. Given a re-emergence of CQ and related analogues as neuroprotectants, it is crucial to understand the underlying mechanism of CQ-induced toxicity to prevent any potential CQ-associated risks to future patients. A small molecule screen to find drugs that induce mitochondrial dysfunction *in vitro* identified CQ and the structurally related 8-hydroxyquinoline (8-OHQ). Their mitochondrial liability, pro-oxidative and cytotoxic activity was subsequently confirmed in some cell lines but surprisingly not in others. Subsequent studies in isogenic cell lines demonstrated that the antioxidant protein NQO1 is differentially expressed in the cell lines tested and potently protects against CQ toxicity. CQ-induced reduction of cellular ATP levels, increased lipid peroxidation and elevated cell death was also attenuated by antioxidants, implicating oxidative stress as the core mechanism of CQ-induced toxicity. These *in-vitro* findings were replicated in zebrafish. Visual acuity in zebrafish larvae that do not express NQO1, was reduced by CQ in a dose-dependent manner, while CQ did not affect visual function in the adult zebrafish that express NQO1. Similarly, pharmacological inhibition of NQO1 activity resulted in CQ-induced oxidative stress in the retina and severe acute systemic toxicity in the adult fish. Given the much higher prevalence of the inactivating C609T NQO1 polymorphism in the Japanese population compared to the European population, the results of this study could for the first time indicate how the geographic restriction of SMON cases to Japan could be explained. Importantly, if CQ or its derivatives are to be used safely for the treatment of neurodegenerative diseases, it seems imperative that NQO1 levels and activity of prospective patients should be ascertained.

## Introduction

The quinoline derivative clioquinol (CQ) was widely used as a topical disinfectant for skin conditions and as an oral antibiotic against diarrhoea. However, the oral formulation was taken off the market in many countries in 1970 after it was linked to about 10,000 cases of subacute myelo-optic neuropathy (SMON) in Japan. SMON was characterised by optic neuritis and axonopathy of the spinal cord. Although a clear mechanistic connection is still disputed, a clear reduction in SMON cases was witnessed following drug withdrawal from the market. A large body of literature supports the presence of CQ toxicity in many experimental systems (Mao and Schimmer, 2008) with the common pathology of SMON-associated neurotoxicity successfully reproduced in CQ-treated animals, which supports a direct connection.

CQ is a lipophilic chelator of iron (Fe), zinc (Zn) and copper (Cu) and is believed to cause cytotoxicity due to its metal chelation and ionophore activity (Arbiser et al., 1998; Mao and Schimmer, 2008). This property effectively transports large amounts of chelated metal ions into cells. Although CQ toxicity was also reported to be independent of metal ions, typically metal-dependent CQ toxicity is increased by the presence of enhanced cation levels, which would suggest that CQ-metal chelates themselves represent the toxic component (Arbiser et al., 1998). However, other explanations have also been proposed. For example, reduced concentrations of available metal ions could impair the functionality of some cellular proteins that require metal ions as essential co-factors (Yagi et al., 1985; Benvenisti-Zarom et al., 2005), such as the antioxidant protein superoxide dismutase (SOD). Indeed, CQ was previously reported to be neurotoxic by a mechanism that included SOD inhibition, based on the ability of CQ to chelate Cu and Zn ions that are essential for SOD activity (Kawamura et al., 2014). In addition to its effect on antioxidants, CQ was also reported to inhibit the 20S proteasome by Cu-dependent and independent mechanisms that increase the accumulation of misfolded proteins, which subsequently can result in cell death (Schimmer, 2011). Furthermore, a metal-independent mechanism of CQ neurotoxicity was proposed that involved the induction of DNA double-strand breaks (Katsuyama et al., 2012). Finally, altered neuronal growth factor signalling was suggested to explain CQ-induced neurotoxicity. In neuronal cells, CQ dose-dependently inhibited nerve growth factor (NGF)-induced Trk autophosphorylation, inhibited NGF-induced mitogen-activated protein kinase (MAPK) phosphorylation and neurite outgrowth (Asakura et al., 2009).

However, none of those modes of toxicity explain the geographic restriction of SMON cases to Japan. Even at the time when oral CQ was removed from the market, it was proposed that a specific genetic background might be responsible for the Japanese SMON cases in clioquinol users (Kono, 1971). Only recently, ATP transporters were suggested as possible targets. A screen to identify compounds that block active cAMP efflux identified clioquinol (Perez et al., 2016). What makes this class of targets attractive is that several function-modifying polymorphisms are highly present in Japan but not in other geographic areas (Perez et al., 2019). It was therefore proposed that a combination of impaired transporter function and transport inhibition by CQ, could trigger SMON pathology, while it could also explain the restriction of SMON to Japan (Perez et al., 2019). However, clinical data suggests that ABCC4 and ABCC11 are not associated with the development of SMON (Matsumoto et al., 2022).

The present study identified the early response protein NADPH-quinone-oxidoreductase 1 (NQO1) not only as target but also as a determinant of CQ-induced toxicity *in vitro* and *in vivo*. NQO1 is a cytoplasmic FAD-dependent flavoprotein that catalyses a two-electron reduction of quinones, quinoneimines, nitroaromatic, glutathionyl-substituted naphthoquinones, di-chlorophenol indophenol (DCPIP) and azo dyes using NAD(P)H as electron donor. For example, this reduction prevents the formation of reactive semiquinones, which would otherwise result in oxidative stress (Dinkova-Kostkova and Abramov, 2015). In addition to its catalytic role to detoxify xenobiotics and quinone redox-chemistry, NQO1 can also directly scavenge superoxide radicals and protect against oxidative stress (Ross and Siegel, 2021). Intriguingly, NQO1 also connects CQ-toxicity at the level of proteostasis. While NQO1 acts as a regulatory gate keeper of the 20S proteasome, CQ is known to inhibit proteasomal function (Dinkova-Kostova and Talalay, 2010; Schimmer, 2011; Ross and Siegel, 2021). NQO1 is highly expressed in heart, liver, lung, breast, colon, vascular endothelia, and adipose tissue. Importantly, considering the pathology of SMON, it is also highly expressed in the eye, within the cornea, lens epithelium, retinal pigment epithelium, optic nerve and nerve fibre tissue (Ross et al., 2000). In addition, NQO1 inactivation has been linked to many pathological conditions including cardiovascular diseases, cancer, metabolic disorders and several neurodegenerative diseases including Alzheimer’s disease (Zhu and Li, 2012; Chhetri et al., 2018).

Somewhat surprising, despite its widely reported toxicity in different models and organisms, CQ and its structural analogues are investigated as disease modifying treatments for neurodegenerative disorders such as Alzheimer’s, Parkinson’s and Huntington’s Disease, based on its metal chelating activity, with some positive preclinical results (reviewed by Perez et al., 2019). Considering the re-emergence of CQ and its structural analogues, it is crucial to understand the underlying mechanism of CQ-induced toxicity beyond the scope of SMON to prevent any potential CQ-associated risks to future patients.

## Methods and materials

### Cell culture

Human hepatocellular carcinoma cells (HepG2, EACC), human embryonic kidney cells (HEK293, EACC) stably transfected with pCI-neo expression plasmid (Promega) containing the sequence for recombinant human NQO1 and rodent neuronal RGC5 cells (provided by Associate Professor Ian Trounce; Centre for Eye Research Australia, Melbourne, Australia) were cultured under standard conditions (37 °C, 5% CO_2_, 95% humidity) in Dulbecco’s Modified Eagle’s Medium (DMEM) containing 1 g/L glucose or in glucose-free DMEM supplemented with either 5% or 10% heat inactivated fetal bovine serum (FBS; Gibco, Life Technologies Victoria, Australia), 100 units/ml penicillin and 100 μg/ml streptomycin (Gibco, Life Technologies Victoria, Australia). In addition, glucose free media was supplemented with 1 g/l d-(+)-galactose and 1 mM sodium pyruvate for some experiments. G418 (0.5 mg/ml stock solution) was used for transfected HEK293 cells every fourth passage to maintain stable cell lines. All cells were routinely passaged every three to 4 days when cell density reached around 80%.

### Colony formation

Colony formation of cells was performed using standard techniques. Cells were seeded at 200 cells per 100 mm cell culture Petri dish (for HEK293 and RGC5 cells) and 1,000 cells/well (for HepG2 cells) in six well culture plates in culture media containing low glucose. After 24 h, cells were treated with different concentrations of CQ (RGC5/HepG2: 0, 1, 2, 3, 4, 5, 10 μM; HEK293: 0, 1, 5, 6, 8, 10 μM) and 8-HQ (RGC5: 0, 1, 2, 3, 4, 5, 10 μM) and incubated 7 days for RGC5 cells, 10 days for HEK293 and 15 days for HepG2 cells due to the different growth rates of these cell lines. After fixing with 2% paraformaldehyde and staining with Coomassie dye, colonies ≥50 cells were scored manually using a standard light microscope. Colony formation was expressed as percentage compared to the untreated control cells. Colony formation was performed as four replicates per experiment.

### Cellular ATP levels

Cellular ATP was detected as described previously (Haefeli et al., 2011). Briefly RGC5, HepG2, HEK293 cells were seeded at 1.5*10^4^ cells/well, 2.0*10^4^ cells/well and 1.5*10^4^ cells/well in 96 well culture plates in their respective culture media. Due to low attachment of HEK293 cells, poly-l-lysine (0.01%) coated 96 well culture plates were used. After 24 h, cells were treated with CQ or 8-HQ at 0, 0.5, 1, 5, 10 μM in glucose or galactose containing culture media for either 24 h (RGC5 and HepG2) or up to 2 h (RGC5, HepG2 and HEK293). After the treatment period, cells were briefly washed twice with 100 μL PBS and lysed with 40 μL of lysis solution (4 mM EDTA, 0.2% Triton X-100) for 5 min at RT. 10 μL of lysate was combined in a white 96 well culture plate with 90 μL of ATP measurement buffer (25 mM HEPES pH 7.25, 600 μM D-luciferin, 75 μM DTT, 6.25 mM MgCl2, 625 μM EDTA and 1 mg/ml BSA, 10 μg/ml firefly luciferase). Luminescence was quantified immediately using a multimode plate reader (Fluroskan Ascent FL Thermo Scientific, VIC, Australia). ATP levels for each individual experiment were averaged from five replicate wells/sample for both the control and each drug concentration and the data were expressed as the percentage of control values. Standardisation of ATP values on protein content/well was only carried out for long-term exposure experiments. ATP measurements were repeated as three independent experiments for all cell types with one representative experiment shown.

### Protein quantification

Quantification of protein content of cell lysates was performed using the DC protein assay kit (Bio-Rad Laboratories PTY Ltd, Gladesville, NSW, Australia) according to the manufacturer’s guidelines. Protein concentrations were calculated using a BSA standard curve.

### Lipid peroxidation

Cellular lipid peroxidation was measured using C_11_-BODIPY_581/591_ (D3861 Invitrogen, Eugene, Oregon, United States) according to the manufacturer’s instructions. Briefly RGC5, HepG2 and HEK293 cells were seeded at a density of 4*10^4^, 5*10^4^ and 5*10^4^ cells/well respectively in black 96 well culture plates. Due to limited attachment, poly-l-lysine (0.01%) coated black 96 well culture plates were used for HEK293 cells. After 24 h the cells were washed once with 100 μL of PBS and were loaded with 100 μM C_11_-BODIPY_581/591_ in HBSS for 30 min under normal culture conditions. Cells were washed once with 100 μL PBS before treated with different concentrations of CQ (0, 0.5, 1, 5, 10 μM) in HBSS for different time intervals (0, 30, 60, 90 and 120 min). After the treatment period, cells were washed once with 100 μL PBS and fluorescence was quantified immediately in 40 μL PBS (Ex/Em 460/535 nm: green fluorescence and 485/600 nm: red fluorescence) using a multimode plate reader (Fluroskan Ascent FL, Thermo Scientific, VIC, Australia). Changes in fluorescence intensity ratio at 535 nm versus 600 nm, indicative of lipid peroxidation, was expressed as a percentage of untreated control values. Lipid peroxidation was measured in at least three replicate wells/sample/experiment. Three independent experiments were performed with one representative experiment shown.

### Western blotting

Proteins were detected by western blotting using standard techniques. Briefly, total cell extracts were prepared using lysis buffer (50 mM Tris pH 7.4, 150 mM NaCl, 2 mM EDTA, 2 mM EGTA, 0.2% Triton X-100, 25 mM β-glycerophosphate, 0.3% NP-40, 25 mM NaF, 0.1% saturated PMSF, 1 μM DTT and 0.1 mM Na_3_VO_4_). Protein extracts were subsequently separated on 12% SDS–PAGE, transferred onto nitrocellulose membrane (GE Healthcare Life Sciences, NSW, Australia) and detected using primary antibodies against NQO1 (ab34173, Abcam, 1:1,000) and GAPDH (ABS16, EMD Millipore, 1:10,000) and species-specific horseradish peroxidase-conjugated secondary antibody (ab97051, Abcam, 1:20,000) for 1 h at RT. Antibody binding was visualized using an image analyser (Chem Smart 5,000) with enhanced chemiluminescence western blotting reagent (ECL; Sigma-Aldrich, Castle Hill, NSW, Australia) according to the manufacturer’s instructions.

### UHPLC-mass spectrometry analysis of CQ

As NQO1 is known to detoxify different xenobiotics such as benzenes and quinones, analysis was performed by combined Ultra-High Performance Liquid Chromatography-Mass Spectrometry (UHPLC-MS) to investigate if CQ is also converted to a potentially less toxic metabolite by NQO1. Four reactions were prepared: 1.) potassium phosphate buffer pH 7.4, CQ 100 μM; 2.) potassium phosphate buffer pH 7.4, CQ 100 μM, NADPH 200 μM; 3.) potassium phosphate buffer pH 7.4, CQ 100 μM, NADPH 200 μM, NQO1 (10 μg/ml); 4.) potassium phosphate buffer pH 7.4, CQ 100 μM, NQO1 (10 μg/ml). The reactions were kept at 37 °C overnight before the mixtures were evaporated under vacuum using a miVac DNA centrifugal concentrator (Genevac, Suffolk, UK) at 50°C for 2–4 h. Any potential conversion of CQ in the presence of NQO1 was measured using UHPLC-MS. Briefly, samples were analyzed using a Waters Acquity H-series UPLC coupled to a Waters Xevo triple quadrupole mass spectrometer. A Waters Acquity UPLC BEH C18 column (2.1 × 100 mm x 1.7 micron particles) was used, with mobile phases A = 0.1% formic acid in water and B = acetonitrile. A linear solvent gradient from 40% A: 60% B to 5% A: 95% B at 8.75 min was used, followed by 3 min re-equilibration time. The flow rate was 0.4 ml/min and the column was held at 45°C. The mass spectrometer was operated in negative ion electrospray mode and CQ was targeted by Multiple Reaction Monitoring (MRM) after the preliminary establishment of an appropriate channel. The ion source temperature was 150°C, the desolvation gas was nitrogen at 1,000 L/h, desolvation temperature was 300°C and the capillary voltage was 2.7 KV. The MRM channel for CQ was m/z 303.9 to 126.95. The cone voltage was 65 V and collision energy was 24 V. Dwell time was 92 ms. Simultaneous full scan data acquisition was carried out from m/z 250 to 500 over 0.3 s with a cone voltage of 60 V. 25 μL samples were injected and data were acquired and processed with Waters MassLynx software.

### Cell-free NQO1 enzyme activity

Activity of human recombinant NQO1 enzyme in the presence of CQ or 8-HQ or the NQO1 inhibitor dicoumarol (Dic) was determined as previously described (Gustafson et al., 2003). Briefly, reaction buffer was prepared in a volume of 100 μL containing 50 mM potassium phosphate buffer (pH 7.4), 0.1% Tween-20, 80 μM DCPIP, 1.25 μg/ml human recombinant NQO1, 200 μM NADPH. The initial reaction mixture was prepared without NADPH, which was added to the reaction buffer to initiate the reaction. Reactions were performed for 1 min at 21 °C in the absence or presence of CQ, 8-HQ or Dic. Enzyme activity was determined by measuring the enzyme-dependent linear decrease in DCPIP absorbance at 600 nm every 1 s over 60 s. The experiment was performed independently three times.

### Cellular NQO1 enzyme activity

Cellular quinone-mediated NQO1 activity in the presence of CQ was determined by conversion of water-soluble tetrazolium dye WST-1 (2-(4-iodophenyl)-3-(4-nitrophenyl)-5-(2,4-disulphonyl)-2H-tetrazolium) (SANTSC-213165, Santa Cruz Biotechnology, United States) as described previously (Tan and Berridge, 2010). Briefly, HepG2 cells were seeded at 1.0*10^4^ cells/well in 96 well culture plates in low glucose culture media. After 6 h, culture media was replaced by challenge media containing glucose-free cell culture media, 2% FBS, 0.3 g/L glucose for 18 h. The cells were then pre-incubated with menadione (10 μM), with or without the NQO1-inhibitor Dic (10 μM), CQ or 8HQ (10, 50, 100 μM) in challenge media for 1 h. After the pre-incubation, the challenge media was replaced by HBSS containing 450 μM WST-1, menadione and Dic or CQ or 8HQ before WST-1 reduction was measured using a multimode plate reader (Thermo Scientific Multiskan GO UV/Vis microplate spectrophotometer, SkanIt Software, VIC, Australia) at 450 nm, 37°C for 120 min. Measurement of cellular NQO1 enzyme activity was repeated independently three times.

### Animal husbandry

All experiments were approved by the Animal Ethics Committee (AEC), University of Tasmania, Australia (Animal Ethics approval numbers: A0012817 and A0015097) and were carried out according to ARRIVE guidelines. The husbandry of adult wild-type zebrafish was carried as previously described (Lawrence, 2007). Fish were raised under standard conditions (14:10 light: dark cycle, 26°C–28˚C) in a recirculating system (Zebtec, Techniplast, United States and Z-hab mini system, AquatiHabitats, Pentair, US for AB). Water parameters were monitored daily and maintained to ensure a predefined target range (pH: 6.5–7.5, conductivity 690–750 μs, bicarbonates 30–35 (mg/L), nitrite: 0 mg/L, nitrate: 0–10 mg/L). Zebrafish were fed twice per day with flake food (Nutrafinmax) in the morning and in the evening and with brine shrimp (hatched in-house from artemia cysts: Inve group) in the morning (3 drops of concentrated brine shrimp per fish from a 5 ml syringe). Breeding of zebrafish was set up fortnightly. Zebrafish were allowed to spawn, and eggs were collected 4 h after the lights turned on at 8 a.m. Fertilized eggs were cultured in embryo media 0.5 x E2 (7.5 mM NaCl; 0.25 mM KCl, 0.5 mM MgSO4, 0.075 mM KH2PO4, 0.025 mM Na2HPO4, 0.5 mM CaCl2, 0.35 mM NaHCO3) in an incubator in the dark at 28.5 ± 0.5°C.

### OKR in zebrafish larvae

The optokinetic response (OKR) in zebrafish larvae was measured using a commercial system (Visio Tracker TSE systems, Germany) using previously described protocols (Huber-Reggi et al., 2013). At five dpf all larvae were transferred in fresh egg media from the treatment plate and OKR were performed from 2 p.m. to 5 p.m. Briefly, larvae were immobilized in pre-warmed 3% methylcellulose solution in 35 mm Petri-dishes. The larvae were well mixed into the methylcellulose mixture and were positioned dorsal side up. Any air bubbles generated during this process were removed using a small needle. The fish were left in methylcellulose for 5–10 min against a white background under flickering light allowing enough time for them to embed. The larvae were exposed to a computer-generated stimulus pattern of rotating black and white stripes. The stimulus used varying absolute velocities (5, 7, 10, 15, 20, 25, 30 deg/sec), constant contrast (10%) and spatial frequency (0.11 cycles/degree) for about 2 min. Before initiation of eye velocity measurements, the fish were pre-stimulated with contrast (10%), spatial frequency (0.11 cycle/deg) and angular velocity (7.5 deg/sec) for a total of 9 s for experiments conducted at varying absolute velocities. Raw eye velocity measurements were filtered for saccades to extract slow-phase velocity. To smooth saccade-filtered eye velocity curves, averages were calculated using a sliding window of seven frames. The average velocities of both right and left eyes at varying stimuli (angular velocities) were calculated in real time (deg/sec) to compare between DMSO- and drug-treated groups.

### OKR in adult zebrafish

The OKR in adult zebrafish was measured according to previously described protocols (Huber-Reggi et al., 2013). Before initiating eye velocity measurements, fish were pre-stimulated for a total 9 s at 99% contrast, 0.2 cycle/deg spatial frequency and 12 deg/sec angular velocity. For the adult fish, both eyes were stimulated with a unidirectional motion stimulus (from right to left) but the evaluation used only in one eye (right) to allow precise positioning of the examined eye. After 48 h of drug exposure, the fish were transferred to a tank containing system water. The fish were briefly anesthetized with 300 mg/l MS-222 and restrained, leaving head and gills free. A single fish was placed in a flow-through chamber supplied with a constant flow of oxygenated water. The water was maintained at 28°C ± 0.5°C and oxygenated using an air pump. The fish were exposed to a computer-generated stimulus pattern consisting of black and white stripes at constant contrast of 99%, 0.2 cycles/degree spatial frequency and at varying absolute velocities of 5, 10, 12, 15, 20 deg/sec for nearly 2 min. Raw eye velocity measurements were filtered for saccades to extract slow-phase velocity. To smooth the saccade-filtered eye velocity curves, averages were calculated using a sliding window of three frames. Average eye velocities of only the right eyes at varying stimuli (angular velocities) were calculated in real time (deg/sec) to compare DMSO- and drug-treated groups.

### Termination of experiment and tissue processing

Larvae and adult zebrafish were euthanized using a rapid cooling technique according to the protocol proposed by the 2013 AVMA (American Veterinary Medical Association) guidelines on euthanasia. Fish irrespective of age were transferred to 2°C–4°C water for at least 20 min. In the case of adult zebrafish, the eyes were removed using a scalpel and fine forceps, whereas for the larvae the whole fish were used. Harvested tissues were either fixed in 4% PFA (in PBS) to carry out histological analysis on paraffin sections and cryosections. Paraffin sections were prepared using standard techniques and Leica ASP 200 auto-processor (Leica Biosystems, VIC, Australia). A microtome (Leica 2,250 microtome, Leica Biosystems, VIC, Australia) was adjusted to 10 μm for coarse sections followed by 4 μm sections when the area of interest was reached. Sections were mounted on IHC microscopy slides (Dako, NSW, Australia) and dried overnight at 37°C. Sections were dewaxed in fresh xylene one and two for 5 min each, 100% ethanol, 95% ethanol, 70% ethanol for 2 min each. Subsequently, the sections were incubated with Mayer’s haematoxylin (H) for 5 min before exposure to ammonia water for 30 s to stain nuclei blue. The sections were counterstained with eosin (E) for 1 min and dehydrated with 95% ethanol for 30 s, two steps of 100% ethanol for 1 min each and finally cleared in two steps of fresh xylene for 2 min each. Mounted sections were examined using a light microscope (Leica DM 2500 microscope, Leica Biosystems, VIC, Australia). Leica Application Suite Version 3.4.1 was used to record the images. All H and E-stained sections of larvae retinas were evaluated for any drug-induced gross morphological changes by comparing the number of retinal ganglion cells (RGC) between DMSO-treated and drug-treated groups. The number of RGC on a 40x image of each retina was counted and the final count was obtained by averaging n = 3 retinas from each group.

### Immunohistochemistry

Paraffin sections were placed in a heater maintained at 60°C for 1 h. After dewaxing and antigen retrieval by heat in 10 mM citrate buffer pH six for 10 min, sections were washed with TBS (Tris base: 50 mM, NaCl: 150 mM pH 7.5) followed by incubation with 3% hydrogen peroxide for 20 min at. The sections were washed three times for 5 min each with TBS-T (TBS +0.05% Tween-20) and blocked with 10% goat serum, 1% BSA, 1% Triton X-100 in TBST 2 h at RT before incubating with primary antibodies against nitro-tyrosine (AB5411-Millipore, 1:400) in blocking buffer (1% goat serum, 1% BSA, 1% Triton X-100 in TBST) overnight at 4°C. After incubation with goat anti-rabbit IgG conjugated to horseradish peroxidase (1:200, ab97051-abcam) or its isotype control antibody (rabbit immunoglobulin fraction, Dako X0903, 1:10,000) in blocking buffer at RT for 1 h, sections were washed with TBS-T and developed with 50 μL of 3, 3′-diaminobenzidine (Abcam, England, UK) in a chromogen solution (1:50) for 5 min. Following washing with TBS-T and water, slides were counterstained with haematoxylin, dehydrated and mounted on IHC microscopic slides. Sections were examined using light microscopy (Leica DM 2500 microscope, Leica Biosystems, VIC, Australia). Leica Application Suite Version 3.4.1 was used to record images.

### NQO1 enzyme activity in adult retina

To prepare retinal cryosections, eyes from adult fish were removed immediately after euthanasia and fixed in 4% PFA (in PBS) for 2 h at 4°C. PFA was replaced by 25% sucrose in PBS until the eyes sank, then replaced with 35% sucrose in PBS and stored overnight at 4°C. Subsequently, the eyes were embedded in cryomolds (10 mm × 10 mm x 5 mm; Tissue-Tek Cryomold, Sakura, United States) containing OCT (Tissue-Tek, Sakura, United States) before freezing down in a for 10 min. The frozen tissue blocks were sectioned into 25 μm thick sections with a cryotome (Leica CM 1850; Leica Biosystems, VIC, Australia) maintained at -20°C. Eye sections were placed on IHC microscope glass slides (Dako, NSW, Australia) and dried for exactly 2 h at RT before NQO1 enzyme activity was detected as previously described with modifications (Murphy et al., 1998). Briefly, the cryosections were washed with Tris-buffer (pH 7.4) and preincubated in solution 1 (25 mM Tris, 0.08% Triton-x, 2 mg/ml BSA) in the presence or absence of 100 μM dicoumarol for 30 min at RT. Solution one was replaced with reaction solution (100 μM NBT (ab146262, Abcam), 1 mM NADPH, 100 μM NQO1 substrate menadione) in the presence or absence of 100 μM dicoumarol and incubated at 37°C for 30 min. Development of the characteristic blue stain was observed under a light microscope before sections were dehydrated and mounted. Images were captured using a light microscope (Leica DM2500; Leica Biosystems, VIC, Australia) at a ×40 magnification. Leica Application Suite Version 3.4.1 was used to record images.

### Statistical analysis

All *in vitro* data are expressed as mean ± standard deviation (SD), as indicated in the figure legends. Statistical significance was performed using Student t-test, one-way or two-way analysis of variance (ANOVA), followed by Dunnett’s multiple comparison tests to differentiate between control and treatment groups. Pearson’s correlation coefficient (r2) was determined for the relationship between two variables when necessary. All *in vivo* data are expressed as mean ± standard error of mean (SEM), as indicated in the figure legends. Statistical significance was assessed using either Student’s t-test or one-way analysis of variance (ANOVA), followed by Dunnett’s multiple comparison tests to differentiate between control and treatment groups. Statistical analysis was performed using GraphPad Prism (Version 6, GraphPad Software Inc, CA, United States). In all assays *p* < 0.05 was considered statistically significant.

## Results

### *In vitro* toxicity of CQ

**FIGURE 1:**
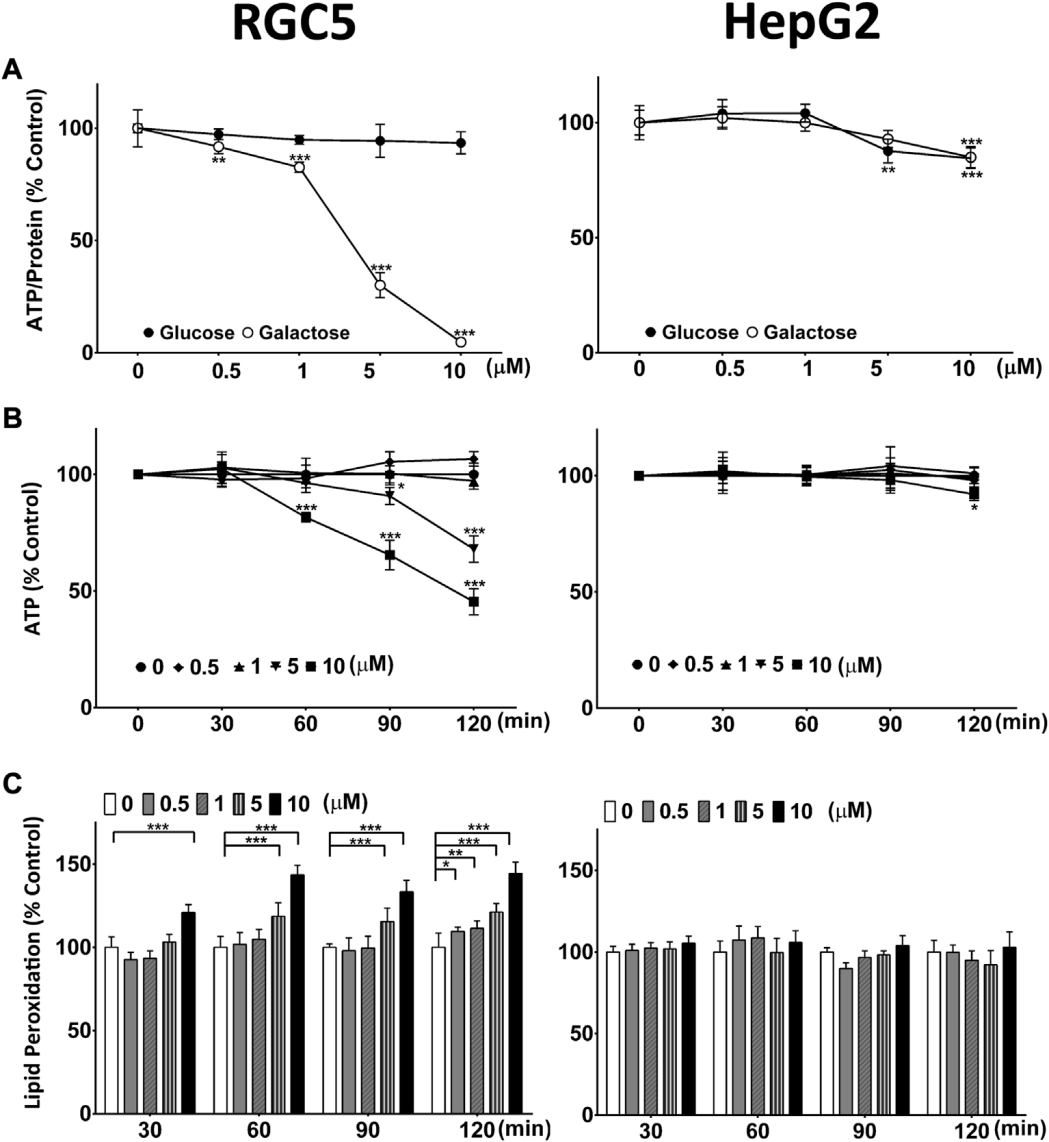
Clioquinol (CQ) induces mitochondrial dysfunction and oxidative stress. **(A)** *Long-term effect of CQ on cellular ATP levels/protein.* ATP levels of RGC5 and HepG2 cells grown for 24 h in glucose- or galactose-containing media in the presence of CQ were measured and standardised on protein content. Data represent the average of at least four or five individual samples per concentration of one experiment out of three independent experiments. **(B)** *Short-term effect of CQ on cellular ATP levels.* RGC5 and HepG2 cells were incubated in galactose-containing media in the presence of up to 10 μM CQ for 30, 60, 90 and 120 min before ATP levels were measured. Data represent the average of at least four individual samples per concentration of one experiment out of three independent experiments. **(C)** *Lipid peroxidation induced by CQ.* RGC5 cells and HepG2 cells grown in glucose-containing media were incubated with CQ up to 10 μM for up to 2 h before lipid peroxidation was measured using BODIPY-C_11_. Data represent the average of at least four individual samples per concentration of one experiment with three independent experiments. P* < 0.05, p** <0.01, p*** <0.001 versus untreated control using one- or two-way analysis of variance (ANOVA) followed by Dunnett’s multiple comparison tests. Error bars = SD.

A screen of a small range of marketed drugs and drug-like molecules to identify drugs with the potential to induce mitochondrial dysfunction, identified CQ and the structurally closely related 8-hydroxyquinoline (8-OHQ) (data not shown). This mitochondrial toxicity was subsequently confirmed in two different, unrelated cell lines (HepG2 and RGC5) (Figure 1). In the presence of galactose-containing medium, CQ significantly reduced ATP levels in RGC5 cells from 0.5 µM onwards, while in glucose-containing medium no effect of CQ on ATP levels was observed (Figure 1A). In contrast, in HepG2 cells, CQ had no major effect on cellular ATP levels, with no differentiation between the two media types. Only at the highest concentrations, a small but significant reduction of ATP was detected in both media (Figure 1B). Similar results were also obtained for the structurally closely related 8-OHQ (Supplementary Figure S1). Since CQ toxicity was previously associated with inhibition of SOD due to the chelating activity of CQ (Schimmer, 2011), we also monitored lipid peroxidation in the presence of CQ in both cell lines. Consistent with mitochondrial dysfunction and increased levels of Reactive Oxygen Species (ROS), CQ induced lipid peroxidation only in the RGC5 but not HepG2 cells (Figure 1C).

### ROS-induced toxicity of CQ

**FIGURE 2:**
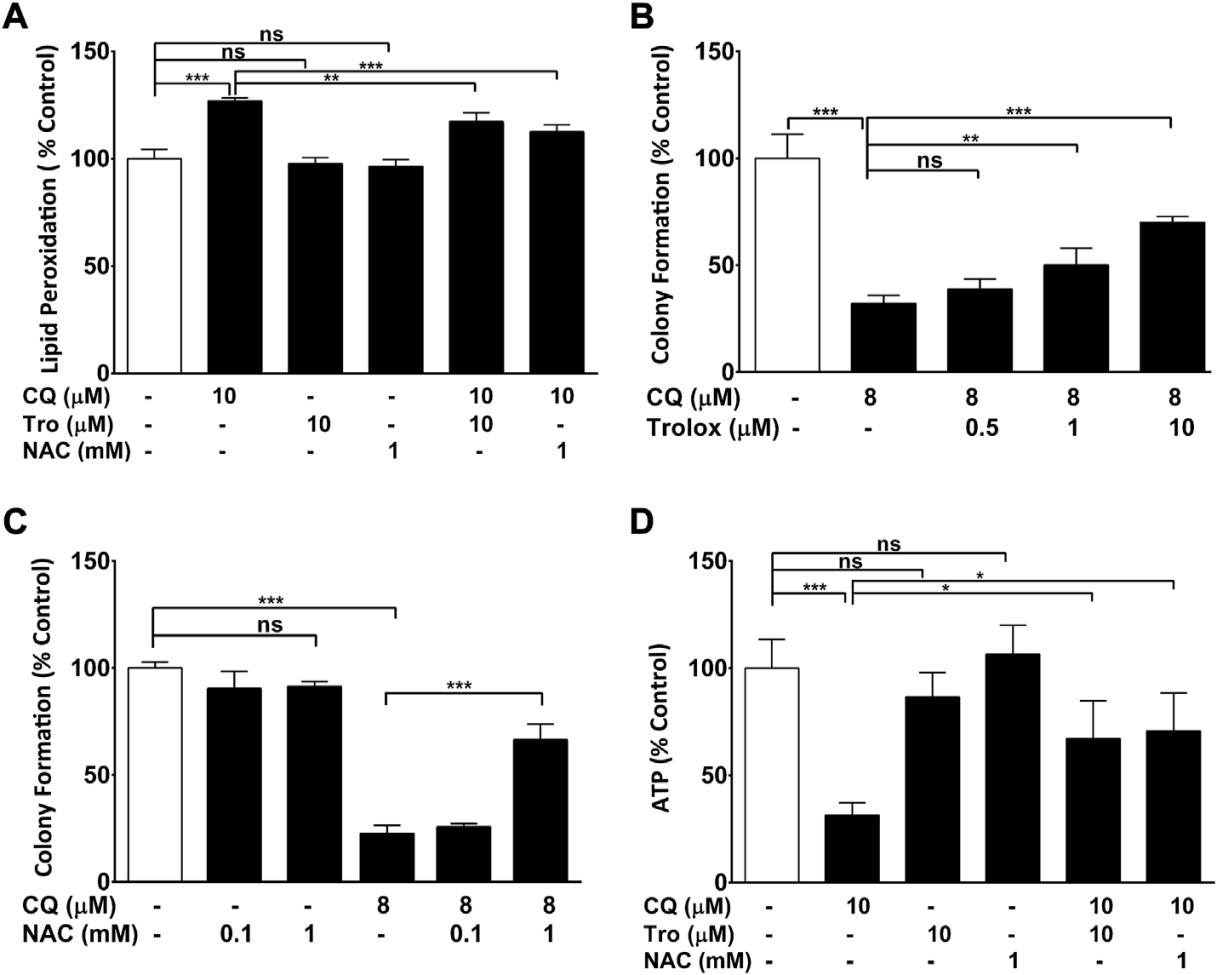
ROS-dependent clioquinol toxicity. *(A*) *Antioxidants ameliorate CQ-induced increase of lipid peroxidation.* **(A)** HEK293-neo cells grown in low glucose were co-treated with antioxidants NAC (1 mM) or Trolox (10 μM) and/or 10 μM of CQ for 2 h before lipid peroxidation was measured using BODIPY-C_11_ dye. **(B, C)** *Antioxidants ameliorate CQ-induced reduction of cellular viability.* HEK293 cells grown in low glucose were treated with CQ and indicated concentrations of **(B)** Trolox or **(C)** NAC or before colony formation was assessed. **(D)** *Antioxidants ameliorate CQ-induced reduction of cellular ATP levels.* HEK293 cells grown in glucose were co-treated with antioxidants NAC (1 mM) or Trolox (10 μM) and/or 10 μM of CQ for 2 h before ATP levels were measured. Data represent the average of at least four individual samples per concentration of one experiment out of three independent experiments. P* < 0.05, p** <0.01 and p*** <0.001 versus control using one-way analysis of variance (ANOVA) followed by Dunnett’s multiple comparison tests. Error bars = SD.

To determine if CQ-induced oxidative stress is a causal factor for CQ toxicity in other cells, the protective activity of antioxidants was evaluated against CQ-induced toxicity in HEK293 cells (Figure 2). Similar to RGC-5 cells, CQ increased lipid peroxidation in HEK293 cells, while the antioxidants N-acetyl cysteine (NAC) and Trolox significant reduced CQ-induced lipid peroxidation (Figure 2A). In these cells, CQ also reduced colony formation (Figures 2B,C) and ATP levels (Figure 2D) consistent with a causal link between lipid peroxidation and these endpoints.

### NQO1-dependency of toxicity

**FIGURE 3:**
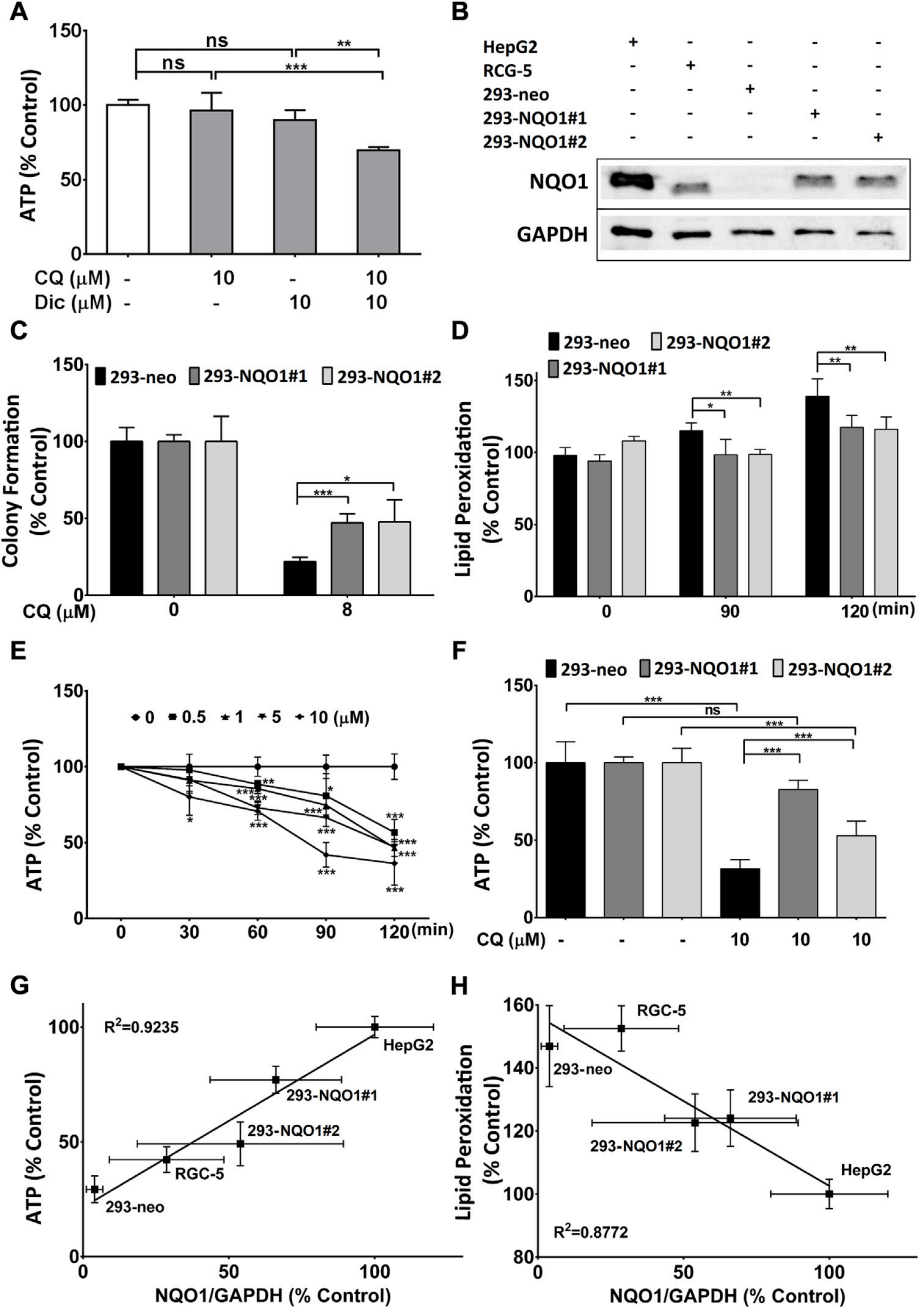
NQO1-dependent clioquinol toxicity. **(A)** *CQ-induced reduction of cellular ATP levels in the presence of the NQ O 1 inhibitor dicoumarol.* HepG2 cells were treated with 10 μM CQ and/or 10 μM dicoumarol (Dic) in galactose-containing media for 2 h before ATP levels were measured. **(B)** *Western blot analysis of NQ O 1 expression.* Representative western blot image of NQO1 and GAPDH in HepG2, RGC5, HEK293-(neo, #1, #2). Data represent one typical experiment out of 5. **(C)** *Cellular viability of HEK293 cells in the presence of CQ.* HEK293 cells were grown in glucose-containing media in the presence of CQ for 10 days. Colony numbers were compared against untreated cells. **(D)** *Effect of NQ O 1 on CQ-induced lipid peroxidation.* HEK293 cells were treated with 10 μM of CQ for up to 2 h and lipid peroxidation was measured using BODIPY-C_11_ dye. **(E)** *Short-term effect of CQ on cellular ATP levels.* HEK29-neo cells incubated in galactose-containing media in the presence of up to 10 μM CQ for 30, 60, 90 and 120 min before ATP levels were measured. **(F)** *NQ O 1 dependent reduction of cellular ATP levels by CQ.* HEK293 cells were treated for 2 h with 10 μM CQ in galactose-containing media before ATP levels were measured. Data represent the average of at least four individual samples per concentration of one experiment out of at least three independent experiments. P* < 0.05, p** <0.01 and p*** <0.001 versus control using Student’s t-test or one- or two-way analysis of variance (ANOVA) followed by Dunnett’s multiple comparison tests where appropriate. Error bars = SD for all experiments. HEK293#1 and #2 = NQO1 overexpressing cells, neo = empty vector. *Correlation of clioquinol (CQ)-induced toxicity* versus *NQ O 1 expression.* Cellular toxicity was measured as changes to **(G)** ATP and **(H)** lipid peroxidation after treatment with CQ (10 μM) for 2 h in galactose-containing media and HBSS respectively. ATP levels were expressed as % CQ-induced ATP reduction in all cell lines relative to HepG2 cells (= 100%). Lipid peroxidation was defined as % increase in lipid peroxidation by CQ in all cell lines relative to HepG2 cells (= 100%). NQO1/GAPDH ratio was expressed as % NQO1/GAPDH in all cell lines relative to HepG2 cells (= 100%). Pearson’s correlation coefficient (r2) was determined for the correlation experiment. Error bars = SD.

Since previous results showed that HEK293 cells express extremely low levels of the antioxidant protein NQO1, while HepG2 cells show high levels of NQO1 expression (Haefeli et al., 2011), we tested if NQO1 inhibition could induce CQ-toxicity in the resistant HepG2 cells (Figure 3A). Indeed, the combination of CQ with the NQO1 inhibitor dicoumarol (Dic) significantly reduced ATP levels, while both compounds on their own did not show any activity (Figure 3A). Based on this data we generated HEK293-based cell lines stably transfected with NQO1 expression plasmids (#1 and #2) or empty plasmids (neo) and compared their NQO1 expression against HepG2 and RGC5 cells. These cells showed significantly different expression levels in the order 293-neo < RGC5 < 293-NQO1#2 < 293-NQO1#1 < HepG2 (lowest to highest expression) (Figure 3B) and were subsequently used to determine if NQO1 expression levels protect against CQ-induced toxicity. In 293 cells, recombinant NQO1 expression was associated with increased colony formation (Figure 3C), reduced lipid peroxidation (Figure 3D) and increased ATP levels (Figure 3F) in the presence of CQ, while in HEK293-neo cells rapidly and dose dependently reduced ATP levels (Figure 3E). To verify the association of CQ toxicity with NQO1 expression, NQO1 protein expression was correlated against CQ toxicity for all five cell lines, where NQO1 expression clearly correlated with CQ-induced reduction of ATP levels (Figure 3G) and lipid peroxidation (Figure 3H).

### NQO1-dependent detoxification of CQ

**FIGURE 4:**
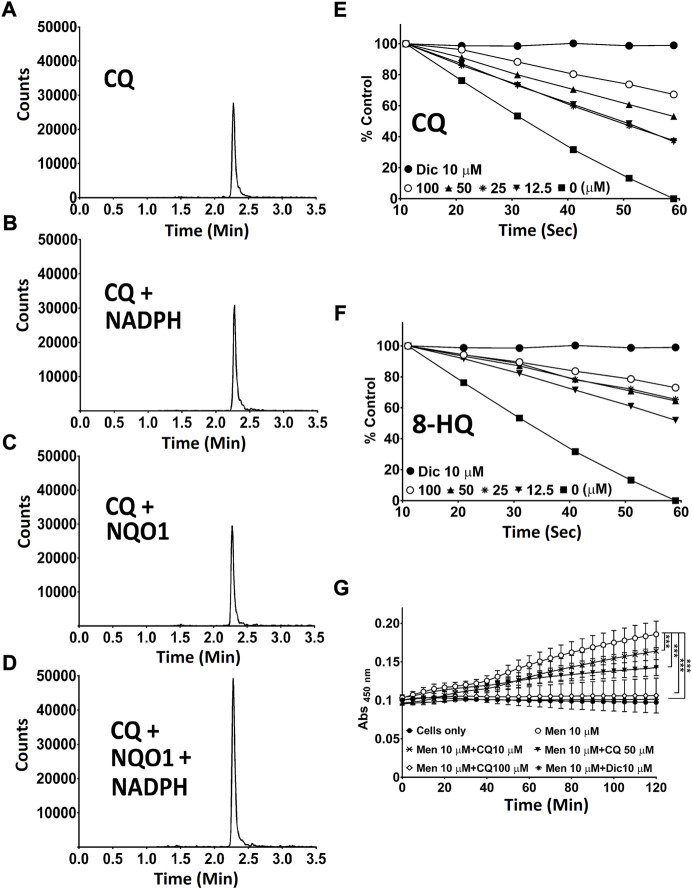
Interaction of CQ and 8-HQ with NQO1. **(A-D)** *Mass spectrometric analysis of CQ.* Potential metabolic conversion of CQ in the presence of NQO1 and NADPH was analysed by mass spectrometry. **(E,F)** *Cell-free NQ O 1 activity measurement using recombinant human NQ O 1.* NQO1 enzyme activity in the presence of 12.5–100 μM CQ **(E)** or HQ **(F)** was determined by measuring the NQO1-dependent linear decrease in DCPIP absorbance at 600 nm. **(G)** *CQ-mediated NQ O 1 inhibition in cells.* Cellular NQO1 enzyme activity in the presence of CQ (10–100 μM) was measured using NQO1-mediated WST-1 dye reduction in HepG2 cells. P* < 0.05, p** <0.01 and p*** <0.001 versus control using two-way analysis of variance (ANOVA) followed by Dunnett multiple comparison tests. Error bars = SD. Men: menadione, CQ: clioquinol, 8-HQ: 8-hydroxyquinoline, Dic: dicoumarol.

One of the best described activities of NQO1, next to its antioxidant function, is the detoxification of quinones to prevent ROS-induced damage (Ross et al., 2000; Ross and Siegel, 2021). Although metabolic conversion of quinolines by NQO1 had not been described previously, such activity could explain our prior observations. Using recombinant human NQO1 in a cell free system, the possible interaction of NQO1 enzyme and CQ was assessed using mass spectrometry. In this system, no degradation or modification of CQ was observed, either by any observable reduction in the targeted channel for CQ (Figures 4A–D), or by the appearance of any new peaks in the non-targeted full scan data channel. In contrast, we detected that both CQ (Figure 4E) and its metabolite 8-HQ (Figure 4F) dose dependently inhibited NQO1 activity in a cell free system as well as in HepG2 cells *in vitro* (Figure 4G).

### Effect of CQ and NQO1 on visual acuity in zebrafish

**FIGURE 5:**
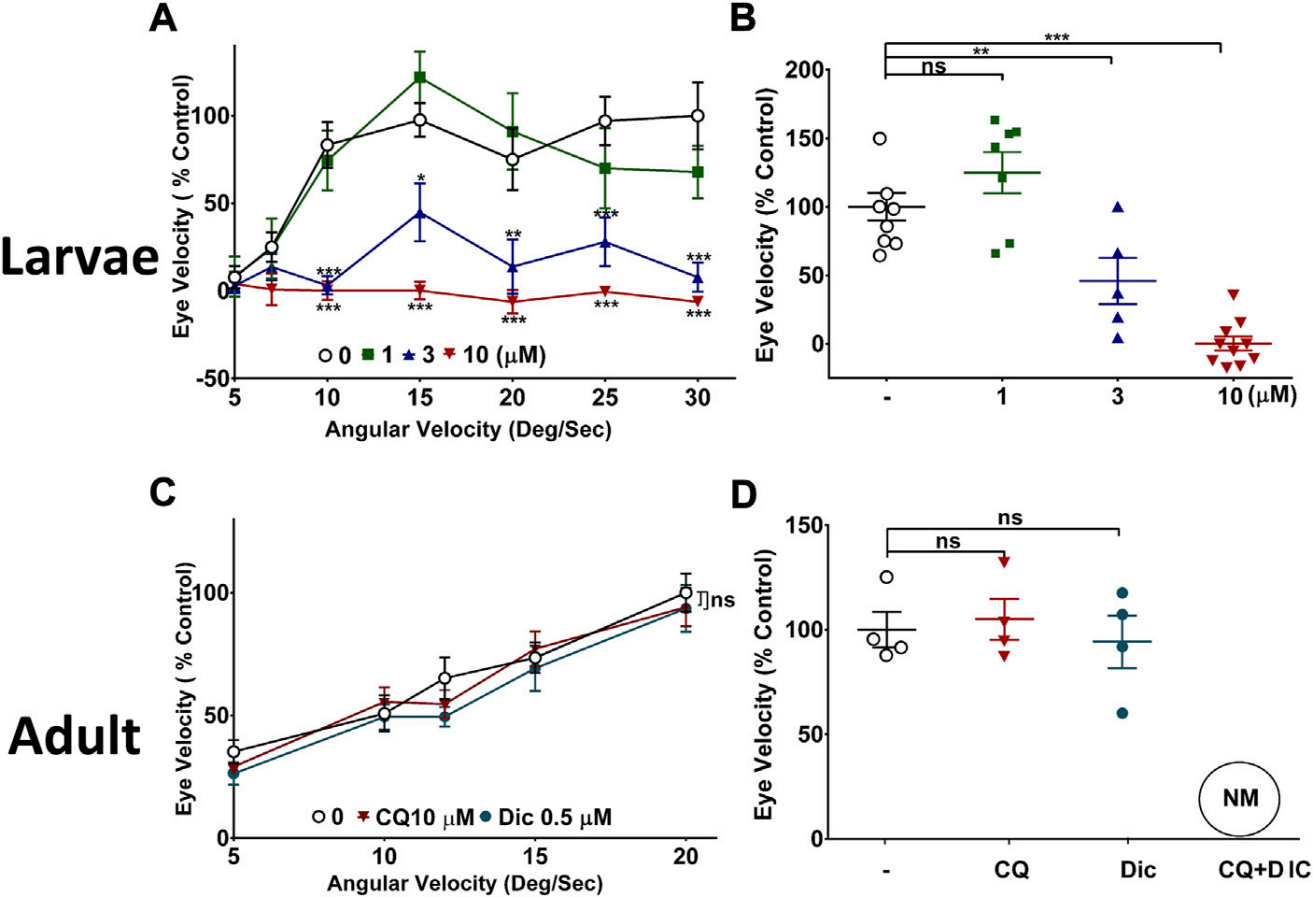
Effect of clioquinol on visual function of zebrafish. **(A,B)** *CQ impairs visual function in zebrafish larvae.* Three dpf PET strain zebrafish were exposed to up to 10 μM CQ for 48 h followed by optokinetic response (OKR) measurement at five dpf at **(A)** varying angular velocities of stimulus **(B)** at one angular velocity of stimulus (15 deg/sec). The visual function was measured in terms of eye velocity under bi-directional motion stimulus. The average velocities of both right and left eyes were used to compare the effect of CQ and is represented as % control. The graph shows the average eye velocity of zebrafish larvae of a typical experiment. **(C,D)** *Effect of CQ on visual function in adult zebrafish.* Adult zebrafish were exposed to 10 μM CQ, 0.5 μM Dic or DMSO for 48 h under 14:10 light: dark cycle conditions before visual acuity was measured. The visual acuity was assessed by measuring eye velocity at varying angular velocities **(C)** and at one angular velocity (15 deg/sec) **(D)** with a unidirectional stimulus. Only the average velocity of right eye was used to compare the effects of CQ. Data represents the average eye velocity the adult zebrafish of a typical experiment. P* < 0.05, p** <0.01 and p*** <0.001 versus untreated control fish using one-way analysis of variance (ANOVA) followed by Dunnett comparison tests. Error bars = SEM, n = 10. CQ: clioquinol, Dic: dicoumarol, NM: not measured.

One of the key pathologies of SMON patients was a loss of visual acuity. Therefore, to study the NQO1 dependency of CQ toxicity on vision, zebrafish were used. Zebrafish are ideally suited for this purpose since NQO1 is not expressed in the retina during embryonic development, while it is expressed in adult fish (Takamiya et al., 2015). Exposure of zebrafish larvae to increasing concentrations of CQ over 48 h significantly reduced visual acuity in a dose dependent manner, with practically no detectable eye movements at 10 µM CQ (Figures 5A,B). Although CQ significantly reduced visual function in larvae treated with CQ ≥ 3 μM, no gross morphological changes were detected at any of the tested concentrations compared to the DMSO-treated control larvae (Supplementary Figure S2). A detailed analysis indicated that, compared to the DMSO-treated group, the numbers of retinal ganglion cells (RGC) were slightly but significantly (88.18% ± 1.74%, *p* = 0.0095) reduced in larvae treated with the highest concentration of CQ (10 μM). In contrast to the larvae, CQ did not affect visual acuity at any concentration in adult zebrafish (Figure 5C). To substantiate the connection between NQO1 expression and CQ toxicity *in vivo*, we aimed to reduce visual acuity in adult zebrafish by inhibiting NQO1 activity. Neither the NQO1 inhibitor dicoumarol (Dic) nor 10 μM CQ administered individually affected visual acuity over a 48h exposure time. In contrast, the combination of CQ and Dic unexpectedly led to the rapid death (NM) of all treated fish within less than 6 h, which prevented visual acuity assessment (Figure 5D).

### Effect of CQ and NQO1 inhibition on retinal NQO1 activity *in vivo*

**FIGURE 6:**
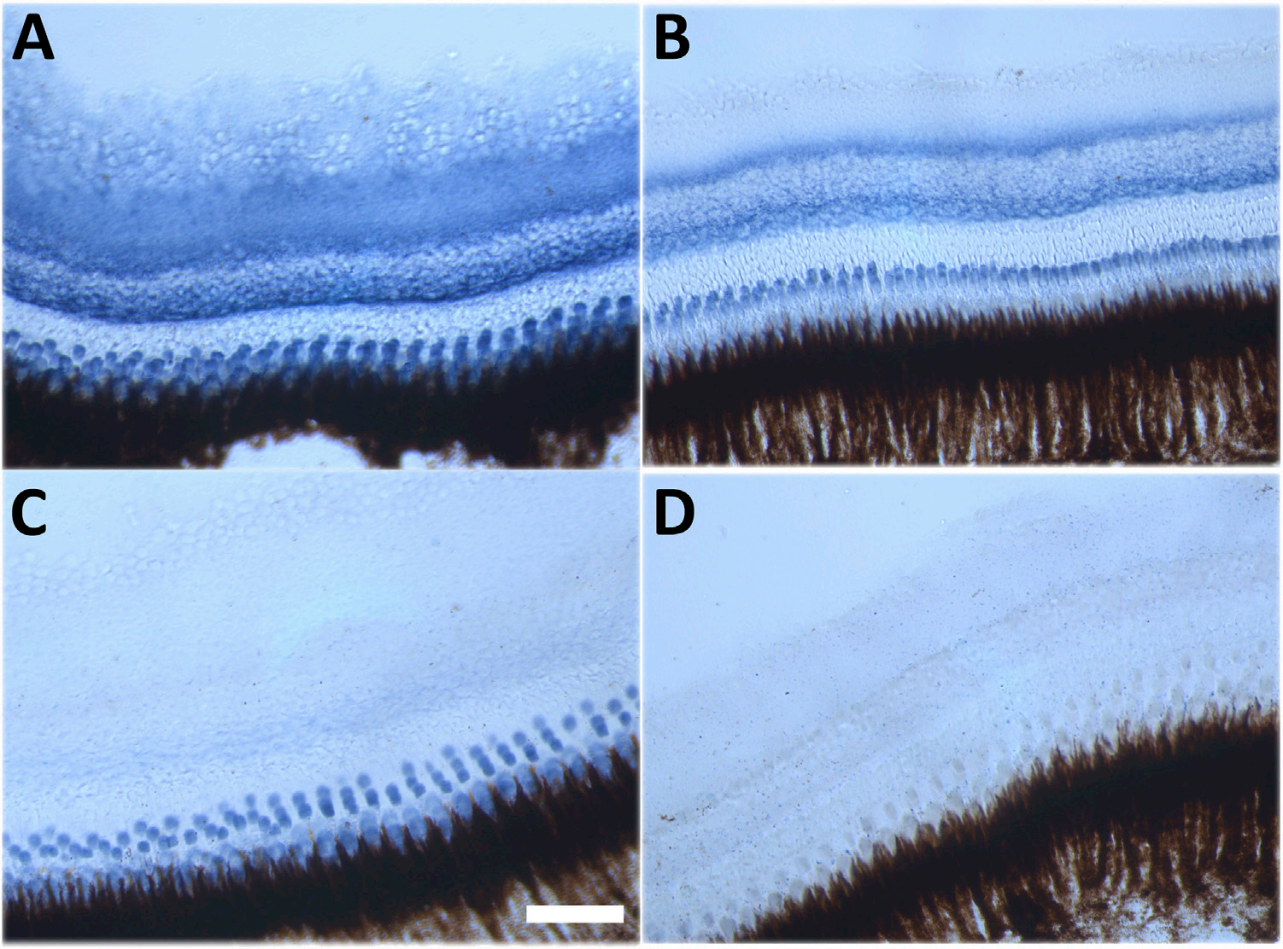
Histochemical detection of NQO1 enzyme activity in the adult zebrafish retina. Representative images of NQO1 enzyme activity in the adult zebrafish retina. Retinal cryosections of adult zebrafish were exposed to 100 μM menadione, 1 mM NADPH, 100 μM NBT for 30 min at 37°C. The presence of NQO1 activity is indicated by a blue reaction product. **(A)** DMSO-treated, **(B)** CQ-treated (10 μM), **(C)** Dic-treated (0.5 μM), **(D)** CQ and Dic-treated (10 μM and 0.5 μM). All *in-vivo* treatments were carried out for 48 h except for the combination treatment where four out of four fish died within 6 h of commencing the treatment. Scale bar = 100 μm. Images were taken at ×40 magnification. CQ: clioquinol, Dic: dicoumarol.

Despite this drastic reaction to the combination of NQO1 inhibition and CQ exposure in adult fish, it was important to detect the effect of both treatments on NQO1 activity *in vivo* (Figure 6). *Ex vivo* zymographic retinal staining demonstrated NQO1 activity (in blue) in most retinal layers with perhaps the exception of the outer nuclear layer (ONL) that only displayed very low activity (Figure 6A). Treatment of fish with CQ alone reduced NQO1 activity in the retinal ganglion cell (RGC), inner plexiform (IP), inner nuclear (IN) and photoreceptor (PR) layers (Figure 6B), while NQO1 inhibition by dicoumarol showed widespread inhibition in most layers with only residual staining in the PR layer (Figure 6C). The combination of both treatments completely abolished NQO1 activity in the retina (Figure 6D).

### Effect of CQ and NQO1 inhibition on RGC numbers

**FIGURE 7:**
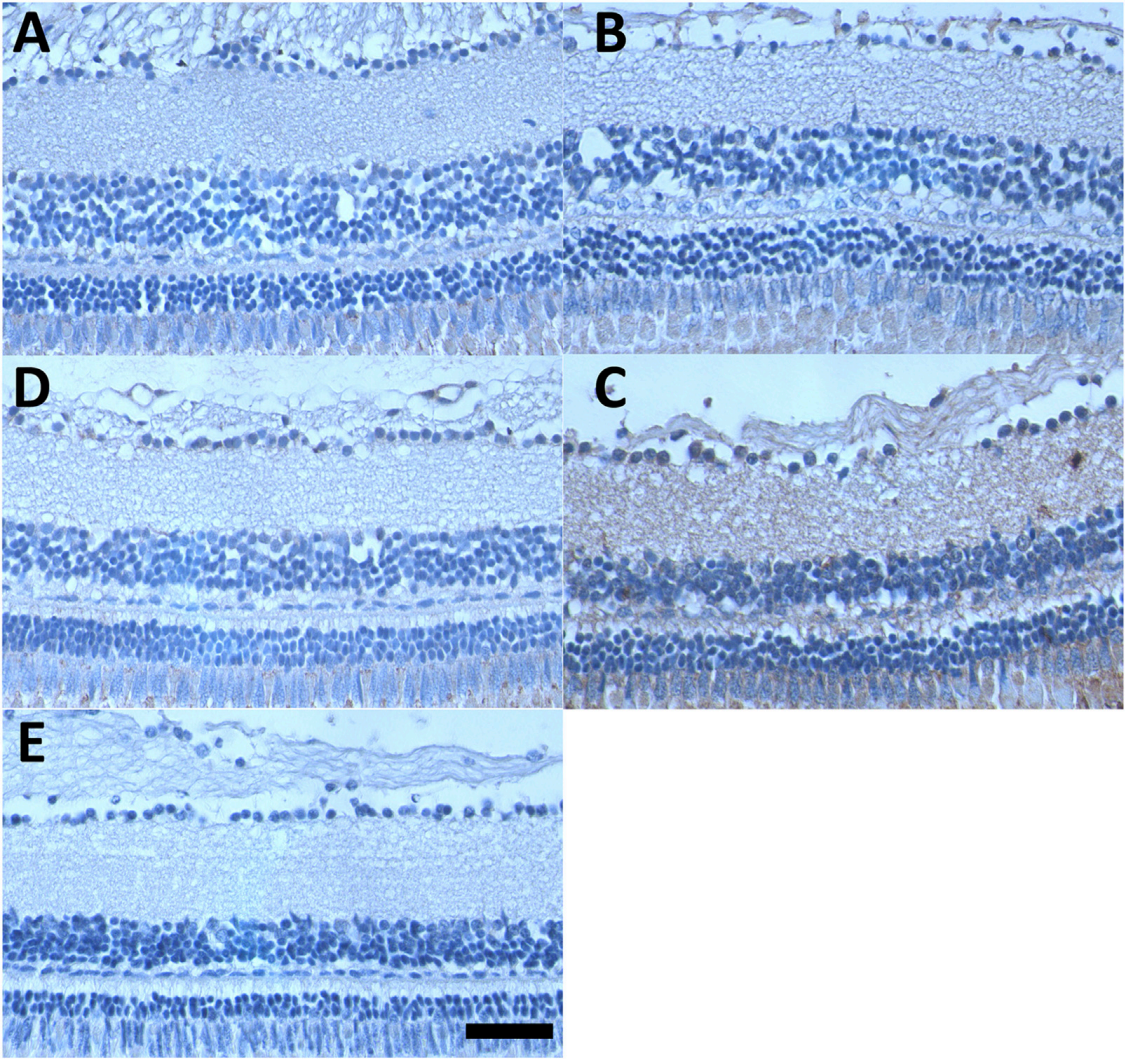
NQO1 prevents CQ-induced oxidative retinal damage. Representative images of nitro-tyrosine staining of the adult zebrafish retina. Formalin-fixed, paraffin-embedded sections of adult zebrafish retina were subjected to immunohistochemical staining (brown colour) for nitro-tyrosine, **(A)** DMSO-treated, **(B)** CQ-treated (10 μM), **(C)** Dic-treated (0.5 μM), *(D*) CQ + Dic-treated (10 μM + 0.5 μM) **(E)** negative control (primary antibody in **(D)** was replaced with non-specific IgG). All the treatments were carried out for 48 h except for the combination treatment (CQ + Dic; **(D)** where four out of four fish died within 6 h of treatment. Scale bar = 100 μm for all images. Images were taken at ×40 magnification. CQ: clioquinol, Dic: dicoumarol.

Since the *in vitro* data of this study strongly suggested that oxidative stress is involved in CQ toxicity, the indicator of oxidative protein damage, nitro-tyrosine, was detected (in brown colour) in the retinas of adult zebrafish (Figure 7). Compared to untreated fish (Figure 7A), individual treatment of adult fish with CQ (Figure 7B) or Dic (Figure 7C) over 48 h did not demonstrate a major increase in oxidative protein damage and was comparable to the negative staining control (Figure 7E). In contrast, the combination of CQ and Dic over ≤6 h markedly increased nitro-tyrosine levels in all layers throughout the retina (Figure 7D).

## Discussion

The results of the present study indicate that NQO1 activity can prevent CQ toxicity *in vitro* and *in vivo,* when NQO1 is inactive, reduced or absent, CQ induces ROS, oxidative damage, mitochondrial dysfunction and cell death. Despite the consistent experimental results, it has to be acknowledged that the mechanism of NQO1 protection against CQ-induced toxicity and the mechanism by which CQ induces oxidative stress remain unclear. The current study identified CQ by its ability to induce mitochondrial dysfunction *in vitro*. Since mitochondrial dysfunction is well known to be associated with elevated levels of oxidative stress, it could be hypothesised that CQ increases mitochondrial ROS production. However, our results indicate that antioxidants are capable of reducing ROS-induced damage and normalising mitochondrial function. This suggests that elevated ROS levels directly cause reduced mitochondrial function and subsequently reduced colony formation. Consequently, CQ appears to increase ROS in a manner independent of mitochondrial redox metabolism. This hypothesis supports previous reports that proposed CQ-induced ROS production by reducing available metal ion levels, which in turn would inhibit superoxide dismutase (SOD) proteins (Kawamura et al., 2014). It is noteworthy that the cell line used in this study is known to express very low baseline levels of NQO1 (Özcelik, 2022), similar to the RGC5 cells used in the present study, which can explain their sensitivity towards CQ. However, NQO1 protein levels can rapidly increase in response to oxidative stress and xenobiotics (Ross and Siegel, 2021; Özcelik 2022). Since NQO1 itself shows superoxide dismutase activity and has several additional antioxidant functions (Ross and Siegel 2021), it is conceivable that elevated NQO1 levels could compensate for the loss of SOD function. However, the results of the present study indicate that NQO1 itself seems to be a target of CQ inhibition, which is supported by previous studies that characterised specific quinoline derivatives as NQO1 inhibitors (Kadela-Tomanek et al., 2017; Ling et al., 2018). In contrast to SOD proteins, which require metal ions as co-factors, NQO1 activity is not dependent on metal ions but utilises flavin adenine dinucleotide (FAD). Therefore, the detailed mechanism by which CQ inhibits NQO1 remains to be identified. However, if CQ inhibits NQO1, how can NQO1 protect against CQ-induced ROS? One explanation could be the presence of a simple concentration dependency. At low cellular levels of NQO1, CQ potently inhibits not only SOD1 but also NQO1, which inhibits both superoxide detoxification mechanisms, leading to superoxide-induced damage and cytotoxicity. The current literature suggests that ROS are not directly produced by CQ but could represents the inability to detoxify superoxide that is generated by normal cellular redox metabolism. Therefore, at sufficiently high cellular concentrations, NQO1 could detoxify CQ-induced ROS if CQ levels are not raised excessively. This model would require a delicate balance between ROS-producing and consuming mechanisms and might be responsible for the protective effects of NQO1 against CQ toxicity. This possibility is supported by the very small dose-effect range observed in the present study where toxicity is observed *in vitro* from 1 µm to a maximal effect at 10 µM which was replicated in the *in vivo* experiments. It must be acknowledged that CQ-induced ROS production was reported before and recently connected to impaired mitochondrial membrane potential and reduced cellular ATP levels (Mizutani et al., 2021), which supports the results of this study. Mizutani et al. also supported the causal connections observed in the present study that CQ is unlikely to impair mitochondrial function directly, with ROS production as a secondary event. Instead, the results of both studies point towards increased ROS production by CQ as a primary event that subsequently impairs mitochondrial function.

What further complicates our understanding of CQ toxicity is that some reports suggested that CQ-metal chelates and not CQ itself are the source of CQ toxicity (Arbiser et al., 1998). CQ-ferric chelate increased iron uptake into neural cells *in vitro*, associated with increased lipid peroxidation and cell death (Yagi et al., 1985), while CQ-zinc chelate reduced mitochondrial membrane potential in human melanoma cells (Arbiser et al., 1998). While these results could imply a ROS-producing activity by CQ-chelates, it could also suggest that NQO1 might not be directly affected by CQ but instead inhibited by CQ-metal chelates (Schimmer, 2011). Although the present study did not provide experimental proof for this possibility, there is some circumstantial support. NQO1 is well described as a proteasome regulator (Ross and Siegel, 2021), while CQ-metal chelates were shown to inhibit proteasome function (Schimmer, 2011). Given the importance of proteasomal clearance of unwanted proteins to maintain cellular protein homeostasis, it comes as no surprise that even low concentrations of CQ-chelates could inflict substantial stress. This effect could be further exacerbated by a ROS-induced accumulation of oxidatively damaged, misfolded proteins that must be removed by proteasomal degradation. In the test system employed by the present study, the addition of metal ions only marginally increased long-term CQ toxicity (Supplementary Figure S1). Therefore, CQ toxicity likely involves both aspects of toxicity to varying degrees depending on the test system and should be discussed in this, more complex, mechanistic scenario.

Despite the unresolved molecular mechanisms of CQ toxicity, it is important to note that the CQ concentrations (≤10 μM) used in the present study are clinically relevant based on pharmacokinetic studies in animals and humans. In animal models of SMON, peak serum levels of CQ that produced neurotoxicity were approximately 17 μM in monkeys and 46 μM in dogs (Matsuki et al., 1997; Bareggi and Cornelli, 2012). In comparison, CQ was administered orally to SMON patients typically around 1.5 g/day (Egashira and Matsuyama, 1982). In healthy human volunteers that received 3 × 0.5 g CQ/day p. o. over 3 days, peak plasma concentrations of CQ reached 30 μg/ml (98 μM) (Jack and Riess, 1973; Bareggi and Cornelli, 2012), while in another trial plasma levels were reported as 24.87 ± 7.037 μM (Ritchie et al., 2003). Although, these CQ plasma concentrations in human subjects were significantly higher than the concentration that completely inhibited cellular viability in the present study, it must be acknowledged that *in-vitro* toxicity in immortalized cell lines does not necessarily correspond to the observed neuronal toxicity of systemic CQ usage in patients.

Despite its association with neurotoxicity, CQ and its structural analogues have been investigated as disease modifying treatments for neurodegenerative disorders such as Alzheimer’s, Parkinson’s and Huntington’s Disease. Considering the re-emergence of this class of molecules, it is crucial to understand the underlying mechanism of CQ-induced toxicity beyond the scope of SMON to prevent potential CQ-induced adverse effects.

The present study provides pre-clinical data that the presence/activity of NQO1 can modify CQ toxicity. This finding has the potential to explain the continuing mystery why SMON cases were restricted to Japan. One reason for reduced NQO1 activity in the general population are NQO1 polymorphisms (Chhetri et al., 2018; Ross and Siegel, 2021). The most frequent and best-studied human NQO1 polymorphism is NQO1*2 (C609T or Pro187Ser), which reduces NQO1 protein stability and therefore cellular NQO1 activity (Siegel et al., 1999). Heterozygote carriers (C/T) only show about 50% NQO1 protein and activity compared to carriers of the C/C genotype. Homozygote carriers (T/T) only show very low to undetectable residual NQO1 activity (Siegel et al., 1999). The striking connection of this polymorphism to the Japanese SMON cases is a much higher prevalence of this inactivating C609T NQO1 polymorphism in Japanese and Asian populations compared to African or European populations (Gaedigk et al., 1998). More than 80% of Europeans harbour the normal C/C NQO1 genotype, while nearly 70% of Japanese carry the inactivating C/T or T/T NQO1 genotype. Based on the results of the present study and the higher prevalence of the C609T NQO1 polymorphism in Japan, a much higher susceptibility towards CQ toxicity should be expected. Clinical trials to examine CQ and its analogues as disease-modifying treatments against neurodegenerative diseases were mostly undertaken in Europe and Australia, where most participants would likely carry the stable C/C NQO1 genotype. Based on the results of this study, this trial population therefore may not have been susceptible towards overt CQ-induced pathologies related to SMON, such as motor disorders and visual impairment. Nevertheless, a few cases of neurotoxicity have been reported. In a randomised placebo-controlled clinical trial in AD patients, three out of 16 patients that received CQ and Vitamin B12 for 36 weeks developed impaired nerve conduction, two out of 16 patients complained of leg numbness and one patient developed visual impairment including loss of visual acuity and colour vision loss (Ritchie et al., 2003). It is important to note that neurotoxicity is typically a rather slow process and may take several weeks to develop, which is why these trial participants may not have developed the full neurotoxicity symptoms associated with SMON. Furthermore, due to the overlapping clinical symptoms of CQ-induced neurotoxicity and some neurodegenerative diseases, drug-induced neurotoxicity (motor disorder, visual impairment) would have been difficult to distinguish from the natural progression of the disease itself.

Overall, the current study provides evidence that CQ-toxicity involves ROS production, which, as a secondary event, impairs mitochondrial function. Since mitochondrial dysfunction is a feature of pretty much every neurodegenerative disorder, these results potentially explain the near exclusive neuropathology observed in SMON patients. In addition, this study implicates NQO1, likely in its function as antioxidant protein, to counteract and protect against CQ-neurotoxicity *in vitro* and *in vivo*. While other genes have been suggested before, the high prevalence of the inactivating C609T NQO1 polymorphism in Japan could explain the geographical restriction of SMON to Japan despite global usage of CQ. Nevertheless, at this stage, it cannot be excluded that polymorphisms in other genes also contribute to CQ toxicity. At the same time, based on the data of the current study, it seems imperative that the activity status of the main antioxidant enzymes such as NQO1, SOD, heme oxygenase (HO) and endogenous antioxidants should be considered in patients before treatment with CQ is initiated to prevent adverse events with potentially critical outcomes.

## Data Availability

The raw data supporting the conclusion of this article will be made available by the authors, without undue reservation.
