# Supplementary figures and images for: NQO1 protects against clioquinol toxicity
Source: Front Pharmacol. 2022 Oct 4;13:1000278. doi: 10.3389/fphar.2022.1000278 (PMC9576850; doi:10.3389/fphar.2022.1000278)

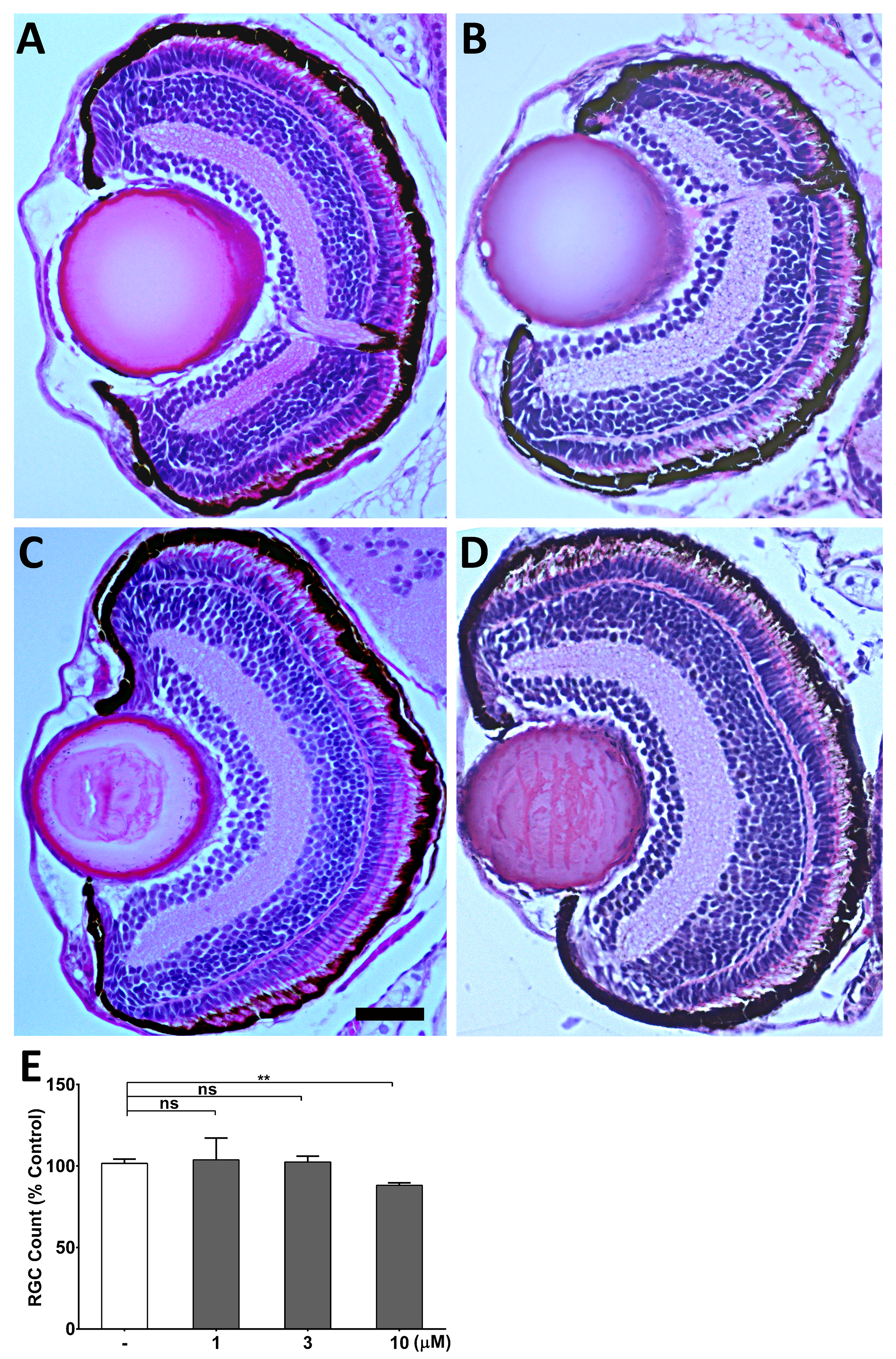

Supplement: Supplementary file 1 [file Image3.TIF]

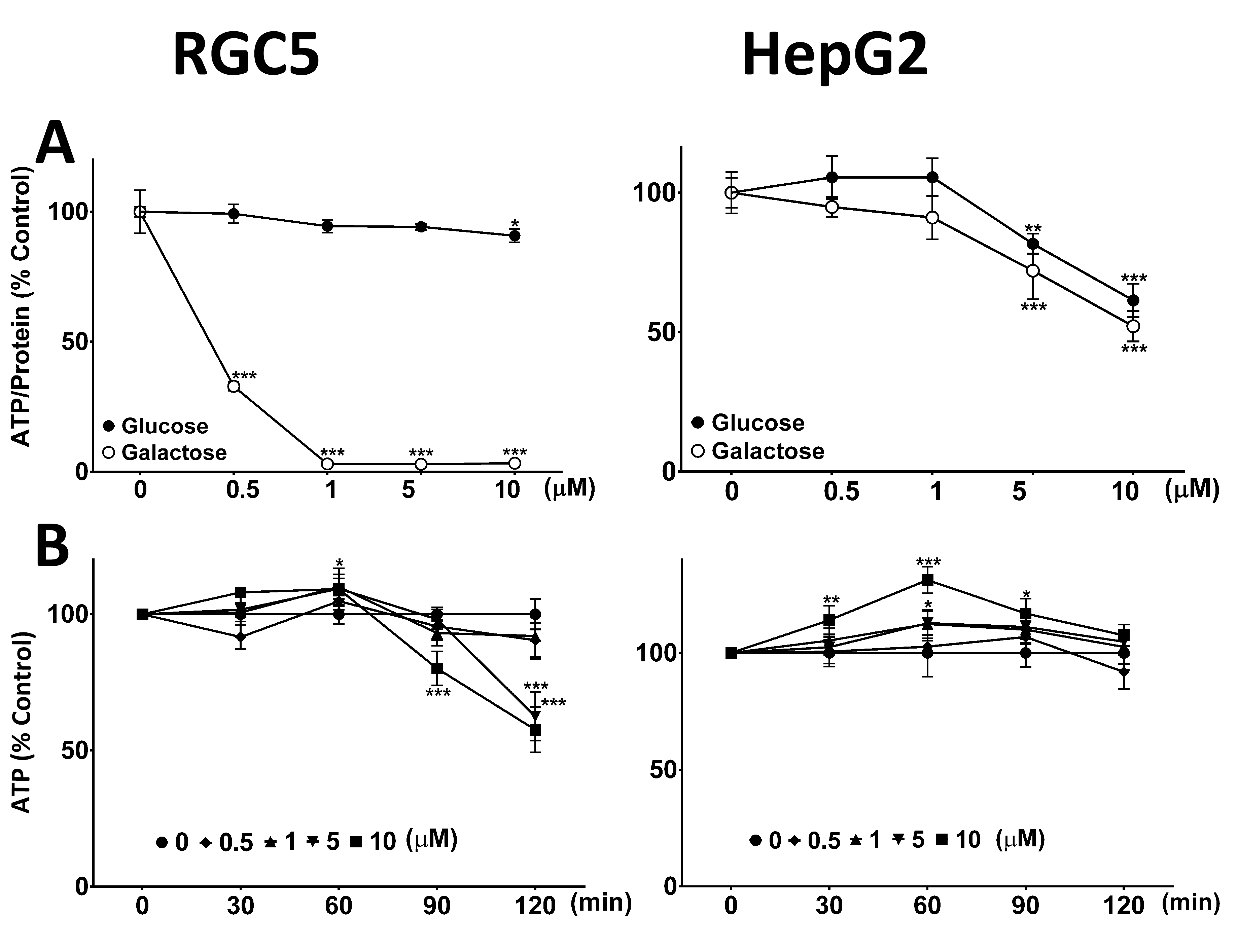

Supplement: Supplementary file 2 [file Image2.TIF]

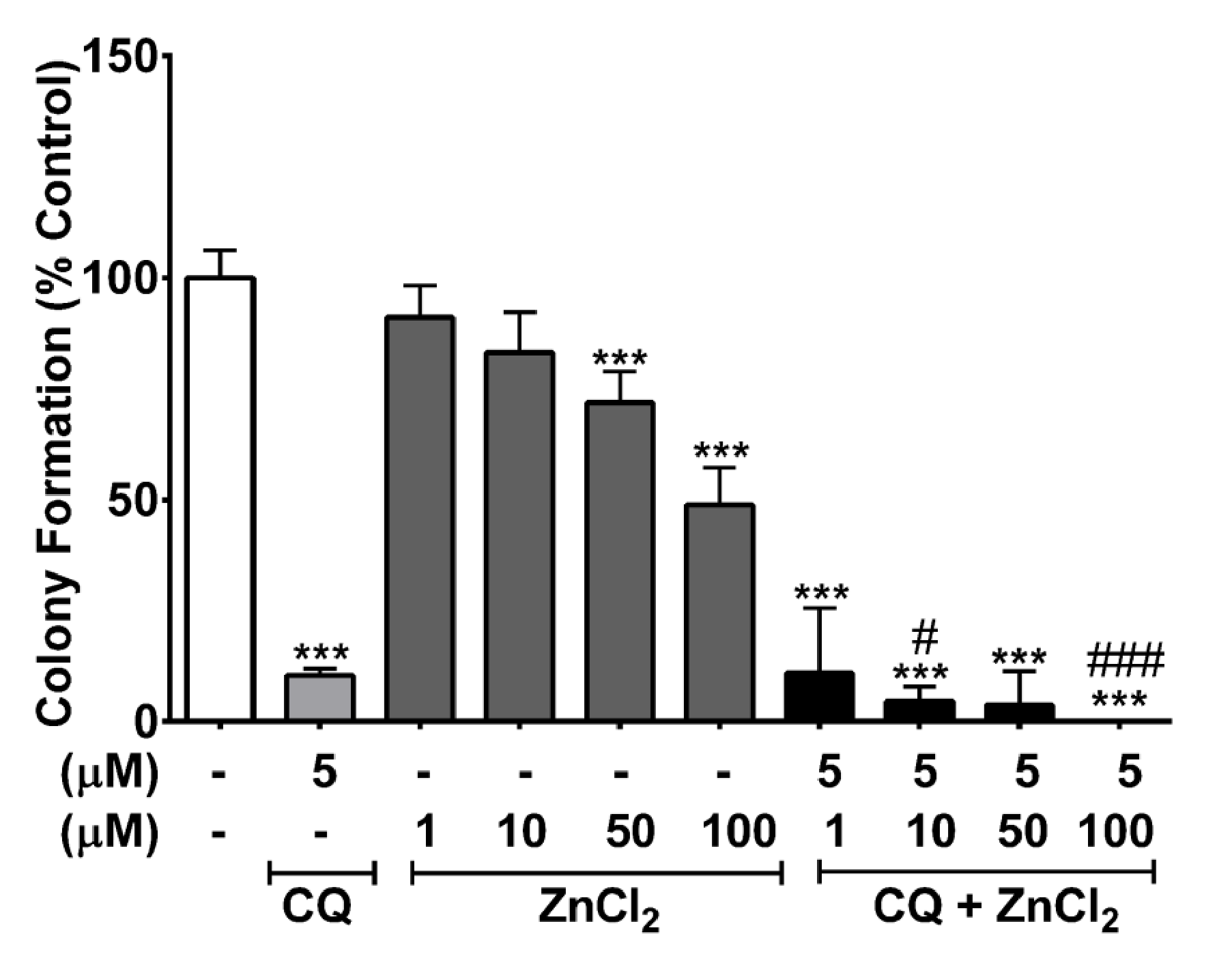

Supplement: Supplementary file 3 [file Image1.TIF]
